# The Value of Different Short-Term Risk Scoring Models in Predicting Long-Term Death of Acute Myocardial Infarction

**DOI:** 10.3390/jcm11175054

**Published:** 2022-08-28

**Authors:** Bosen Yang, Lin Bai, Yike Zhang, Yiheng Cheng, Chunyan Zhao, Baotao Huang, Mao Chen

**Affiliations:** Department of Cardiology, West China Hospital, Sichuan University, Chengdu 610041, China

**Keywords:** risk score, acute myocardial infarction, long-term prognosis

## Abstract

Background and aims: To compare the value of three commonly used cardiovascular short-term risk scoring models, the GRACE score, TIMI score, and HEART score, in predicting the long-term prognosis of patients with acute myocardial infarction. Methods: The hospitalization data of patients who were hospitalized in West China Hospital of Sichuan University from 2011 to 2013 and diagnosed with acute myocardial infarction (AMI) were collected. The patients were scored by GRACE score, TIMI score, and HEART score. The long-term follow-up of patients was conducted until the end of January 2021. All-cause death and time of death of patients were confirmed by telephone follow-up, electronic medical record query, and household registration information. The predictive ability of different risk scores for long-term prognosis was compared according to the receiver operating characteristic (ROC) area under the curve (AUC), and the ability to distinguish patients with different risk levels was compared according to Kaplan–Meier survival curves. Results: The study ultimately included 2220 patients, with a median follow-up of 8 years and 454 (20.5%) deaths until the end of follow-up. Whether in ST-segment elevation myocardial infarction (STEMI) patients or non-ST-segment elevation myocardial infarction (NSTEMI) patients, the AUC value of the GRACE score (both AUC = 0.734) was significantly higher than the TIMI score (AUC = 0.675, *p* < 0.01; AUC = 0.665, *p* < 0.01) and HEART score (AUC = 0.632, *p* < 0.01; AUC = 0.611, *p* < 0.01) until the end of follow-up. In terms of risk stratification, the Kaplan–Meier survival curve shows that both THE GRACE score and TIMI score can distinguish AMI patients with different risk levels (*p* < 0.01), but the risk stratification ability of the HEART score in AMI patients was poor (*p* > 0.05). Conclusion: The GRACE risk score could represent a more accurate model to assess long-term death of acute myocardial infarction, but further studies are required.

## 1. Introduction

Cardiovascular disease is the leading cause of death worldwide. In Europe, more than 4 million people die each year from cardiovascular disease, of which nearly 1.8 million die from coronary artery diseases, accounting for 20% of all deaths in Europe [1]. Of all coronary artery diseases, acute myocardial infarction (AMI) is the most serious, with approximately 700,000 Americans experiencing AMI and another approximately 300,000 experiencing recurrent events each year in the United States [2,3,4,5]. In China, the total hospitalization cost of ischemic heart disease in 2018 was USD16.296 billion including 23.567 billion yuan for AMI, and the average annual growth rate of AMI hospitalization cost was as high as 26.89% [6]. Although reperfusion therapy and early pharmacological treatment have led to a significant reduction in AMI mortality over the past 30 years, the AMI in-hospital mortality has remained higher than 4% until now [2,3,4,5]. Mortality in AMI patients is associated with many factors, and understanding the associated risk factors can help develop clinical strategies and provide valuable prognostic information to clinicians and patients. Researchers have developed many risk scoring models that are based on different risk factors over the past 20 years, and risk scoring models can help clinicians to risk stratify patients and predict the risk of death and/or the incidence of adverse events in patients [7]. Currently, risk scoring models have become an important part of clinical work and the 2018 ESC guidelines also recommend the use of risk scores for risk stratification of AMI patients [8].

The GRACE score, the TIMI score, and the HEART score are three of the most widely used scoring models [9,10,11,12]. The GRACE score focuses on in-hospital deaths of patients and its area under the curve (AUC) for predicting in-hospital death reached 0.83 and 0.84 in the model building and validation cohorts, respectively [9]. The TIMI score has two different risk models for patients with non-ST-segment elevation infarction (NSTEMI) and patients with ST-segment elevation infarction (STEMI). The TIMI score for NSTEMI predicts a composite endpoint event consisting of all-cause death, new or recurrent MI, and urgent revascularization within 14 days and has been validated in other cohorts [10,13]. The TIMI score for STEMI has an AUC value of 0.78 for predicting 30-day death and has been validated in community populations, with similar predictive accuracy in patients that are undergoing percutaneous coronary intervention (PCI) [14]. The HEART score focuses on the risk of major adverse cardiac events and death in the short term, with endpoint event rates of 2.5%, 20.3%, and 72.7% for patients that are at low, intermediate, and high-risk, respectively [12]. Its predictive power has been validated in two other studies [15,16].

In addition to the three scores that are mentioned above, other cardiovascular risk scoring models that are widely used in clinical practice also focus mostly on the in-hospital mortality and short-term adverse event rates of patients [7]. However, in addition to the short-term prognosis, the more distant prognosis of AMI also deserves our attention. Data from the ESC national registry show that the average in-hospital mortality rate of STEMI patients is approximately 5%, while data from the angiographic registry show that the 1-year mortality rate of STEMI patients reaches 10% [17,18,19]. The CHINA PEACE study showed that the in-hospital mortality rate of Chinese STEMI patients is approximately 7–9% and a 1-year mortality rate of up to 28% [20]. All of these findings suggest the importance of assessing the long-term prognosis of AMI patients. Although there are some risk scoring models that can be used to predict the long-term prognosis of patients with coronary artery disease, such as the PAMI score and the CADILLAC score, the former excludes patients with cardiogenic shock, recent stroke, end-stage renal failure, and a life expectancy <1 year, and the latter excludes patients with cardiogenic shock, complex coronary anatomy, and emergency PCI, and therefore, does not reflect the true characteristics of this patient population and is not widely used in the clinic [21,22]. Therefore, assessing the value of the short-term risk scoring model, which is currently widely used in clinical practice, in predicting the long-term prognosis of AMI patients may be a more convenient and rapid approach, while previous studies in this area are very limited, and all suffer from small study samples, short follow-up periods, and inconsistent findings [23,24,25,26].

Therefore, the purpose of this study was to evaluate and compare the accuracy and risk stratification performance of three clinically widely used short-term risk scoring models, the GRACE score, the TIMI score, and the HEART score, in predicting the long-term death of patients with AMI.

## 2. Methods

### 2.1. Study Population

This study was approved by the Clinical Trials and Biomedical Ethics Committee of West China Hospital of Sichuan University, No. 2012 (243). The West China Hospital of Sichuan University is a national-level medical center in western China. It has 2 medical centers and 4300 beds. The annual number of outpatient and emergency department visits is 7.75 million, with 283,000 discharged patients and 196,000 surgeries. Patients with acute myocardial infarction that were hospitalized at West China Hospital of Sichuan University from January 2011 to December 2013 were included retrospectively and consecutively. The inclusion criteria were: (i) age > 18 years, and (ii) diagnosis of “acute ST-segment elevation myocardial infarction” or “acute non-ST-segment elevation myocardial infarction” during hospitalization, with the diagnostic criteria based on the third global definition of myocardial infarction. The exclusion criteria were: (i) the type of infarction diagnosis was unclear, (ii) in-hospital death or automatic discharge due to abandonment of treatment in critical condition, or (iii) death after less than 30 days of follow-up at discharge. All hospitalized AMI patients received standard guideline-based treatment.

### 2.2. Data Collection

The information system of West China Hospital of Sichuan University and the laboratory information system were used to collect the clinical data and laboratory test results of patients. The data included clinical data, past medical history, laboratory tests, and hospital medication. The clinical data included sex, age, body mass index, smoking status, blood pressure, heart rate, Killip classification, clinical diagnosis, and concomitant diseases. The past medical history included a history of previous heart attack, history of previous PCI/coronary artery bypass grafting (CABG), history of cerebrovascular disease, and a history of peripheral vascular disease. Laboratory tests include blood creatinine and troponin T (not high-sensitivity troponin T). Inpatient medications included aspirin, P2Y12 receptor inhibitors, angiotensin-converting enzyme inhibitor (ACEI)/angiotensin II receptor blocker (ARB)/angiotensin receptor neprilysin inhibitor (ARNI), statins, and beta-blockers.

### 2.3. Risk Scores

The GRACE, TIMI, and HEART scores were calculated based on the general condition, clinical history, physical examination, ECG performance, laboratory tests, and risk factors that were collected on admission. The GRACE score contains eight evaluation indicators of age, heart rate, systolic blood pressure, Killip classification, ECG ST-segment performance, serum creatinine concentration, troponin level, and cardiac arrest on admission, with scores ranging from 1 to 363. Patients with STEMI less than 126 are classified as low-risk, 126–154 as intermediate-risk, and greater than 154 as high-risk; patients with NSTEMI less than 109 are classified as low-risk, 109–140 as intermediate-risk, and greater than 140 as high-risk.

The TIMI score of NSTEMI contains 7 evaluation indexes of age, cardiovascular risk factors, history of coronary stenosis, ST-segment performance of ECG, severity of angina, aspirin use, and troponin level, with a score of 0–7, of which 0–2 is low-risk, 3–4 is intermediate-risk, and 5–7 is high-risk. The demographic characteristics, cardiovascular risk factors, comorbidities, and medical history were collected by a questionnaire interview at admission or search in medical records. Data on blood pressure, heart rate, laboratory data, angiographic results, medications, and revascularization therapy were obtained from medical records. The TIMI score for STEMI contains eight evaluation indicators: age, diabetes, hypertension or angina, systolic blood pressure, heart rate, Killip classification, weight, ECG performance, and time to visit, with a score of 0–14, of which 0–3 is low-risk, 4–6 is intermediate-risk, and 7–14 is high-risk.

The HEART score contains 5 evaluation indexes of medical history, electrocardiogram, age, cardiovascular risk factors, and troponin, with a score of 0–10, of which 0–3 is low-risk, 4–6 is intermediate-risk, and 7–10 is high-risk (Table 1).

### 2.4. Follow-Up and Study Endpoint

The endpoint event was all-cause death. The follow-up information was collected from the identification information and contact information that was provided by the patients in the hospital, and follow-up information was obtained through telephone follow-up, electronic medical record query, and household registration information to record the occurrence of the endpoint event of the patients in detail. The follow-up was conducted until the end of January 2021.

### 2.5. Statistical Analysis

SPSS 22.0 statistical software was used for data processing. The measurement data obeying normal distribution were expressed using the mean ± standard deviation, and the independent samples *t*-test was used for comparisons between the groups. The measurement data that did not obey normal distribution are expressed as the median (lower quartile, upper quartile), and the Mann–Whitney U test was used for comparisons between groups. Count data were expressed using the number of cases, rates, or composition ratios, and the χ^2^ test was used for comparison between the groups. The discriminatory ability of the risk scores was assessed by the area under the receiver operating characteristic curve (AUC), and the statistical significance of the difference between the AUCs was tested by the DeLong test. The mortality rates of patients with different levels of risk were analyzed by Kaplan–Meier survival curves and log-rank tests. The Hosmer–Lemeshow test was used to evaluate the calibration ability of the risk models. All the tests were performed using a two-sided test with a test level of α = 0.05.

## 3. Results

**Figure 1:**
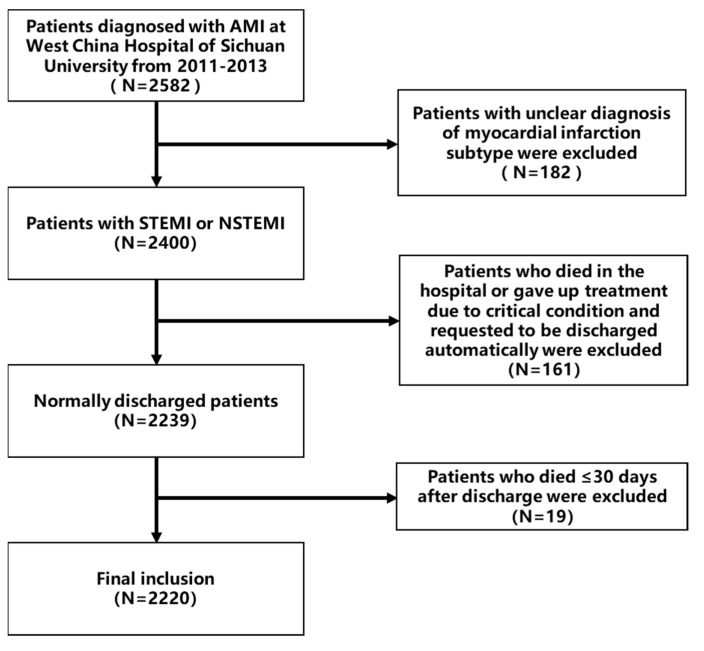
Patient selection process.

A total of 2582 patients with AMI who attended West China Hospital of Sichuan University from January 2011 to December 2013, of whom 182 had an unclear type of infarction diagnosis and 161 died in-hospital or automatically requested to be discharged due to abandonment of treatment for critical illness were excluded. To further reduce the effect of acute infarction critical illness on death in the short-term, the study excluded an additional 19 people who died within 30 days of discharge follow-up (including 30 days), and finally included 2220 people (Figure 1).

The mean age of the patients was 64 years, 1752 were male (78.9%), 49.5% had hypertension, 20% had diabetes, 3% had hyperlipidemia, 61.2% were currently smoking, 11.5% had a history of MI, 1.5% had a history of PCI, and 0.2% had a history of CABG. The median follow-up was 8 years (interquartile range: 7.3–8.9 years) and there were 454 deaths (20.5%). There were 1494 STEMI patients with a median follow-up time of 8.1 years (interquartile spacing: 7.4–9 years), 30 deaths at 1 year (2.0%), 79 deaths at 3 years (5.3%), 135 deaths at 5 years (9.0%), and 236 deaths to the end of follow-up (15.8%); 726 NSTEMI patients with a median 7.9 years (interquartile range: 6.8–8.8 years), 34 (4.7%) deaths at 1 year, 73 (10.1%) deaths at 3 years, 124 (17.1%) deaths at 5 years, and 218 (30.0%) deaths by the end of follow-up (Table 2).

**Figure 2:**
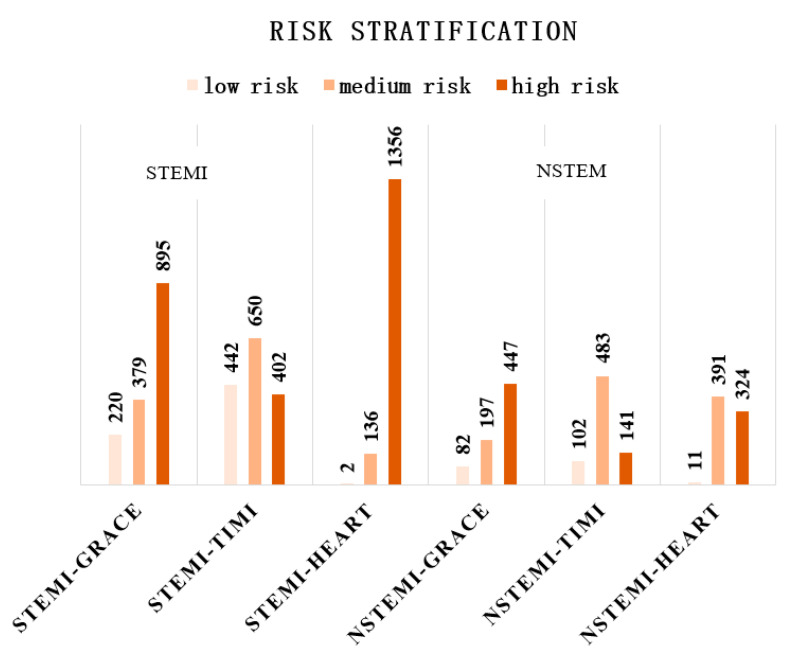
Risk stratification of STEMI and NSTEMI patients in different risk models.

Among the STEMI patients, the median GRACE score was 164 (interquartile range: 136–189), with 60% of patients scoring as high-risk; the median TIMI score was 5 (interquartile range: 3–7), with a predominance of intermediate-risk patients (43.5%); the median HEART score was 8 (interquartile range: 7–9), with 90.8% of patients scored as high-risk. Among the NSTEMI patients, the median GRACE score was 150 (interquartile range: 128–173), with 61.6% of patients still at high-risk; and the median TIMI score was 4 (interquartile range: 3–4), with 66.5% of patients at intermediate-risk; the median HEART score was 6 (interquartile range: 6–7), with approximately half of patients at intermediate-risk and half of patients at high-risk (54% and 45%) (Figure 2).

**Figure 3:**
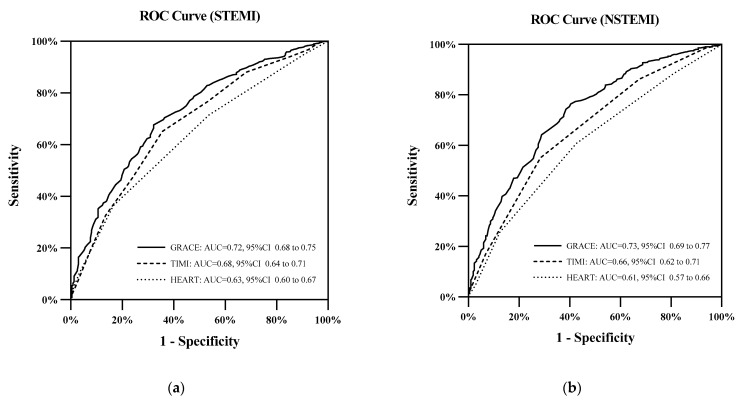
(**a**) ROC curves for the three risk scores in STEMI patients at the end of follow-up. (**b**) ROC curves for the three risk scores in NSTEMI patients at the end of follow-up.

Among the STEMI patients, the AUC values of the GRACE score were 0.775, 0.762, 0.740, and 0.734 at 1, 3, and 5 years and by the end of follow-up, respectively. GRACE had significantly higher AUC values than the TIMI score and HEART score at 3 years, 5 years, and at the end of follow-up (*p* < 0.05). The TIMI score performed slightly worse than the GRACE score, with AUC values of 0.770, 0.719, 0.702, and 0.675 at the four time points, and significantly higher than the HEART score at 3 years, 5 years, and at the end of follow-up (*p* < 0.05). The HEART score performed relatively poorly, with AUC values of 0.719, 0.643, 0.617, and 0.617, respectively. The AUC values for all three scores tended to decrease over time, but the GRACE score was the most stable overall (Table 3, Figure 3a).

In NSTEMI patients, the GRACE score had AUC values of 0.779, 0.762, 0.738, and 0.730 at 1 year, 3 years, 5 years, and at the end of follow-up, respectively. Additionally, the GRACE score had significantly higher AUC values at 3 years, 5 years, and at the end of follow-up than the TIMI score and HEART score (*p* < 0.05). The AUC values of the TIMI score for predicting all-cause mortality were 0.730, 0.678, 0.656, and 0.665 at the four time points, respectively. The AUC values for the HEART score at the four time points were 0.739, 0.658, 0.629, and 0.611, which remained the lowest of the three. However, the difference in the AUC values between the TIMI score and HEART score was statistically significant only at the end of follow-up (*p* < 0.05) (Table 3, Figure 3b).

**Figure 4:**
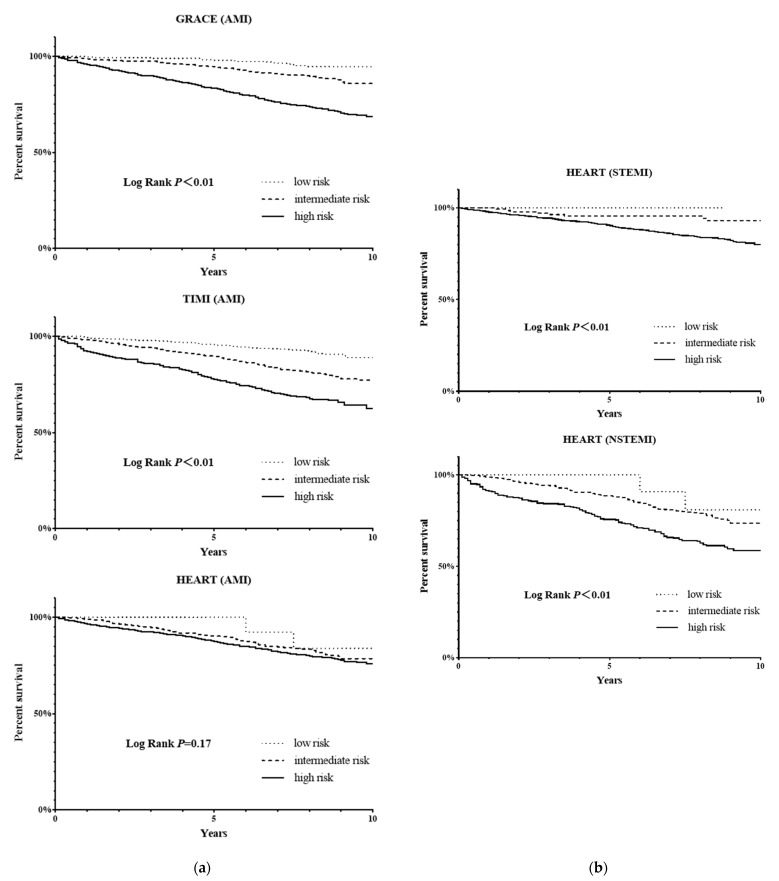
(**a**) Kaplan–Meier survival curves for AMI patients in three risk scores. (**b**) Kaplan–Meier survival curves in patients with STEMI or NSTEMI in the HEART score.

The Kaplan–Meier survival curves showed that among all the AMI patients, the GRACE score and TIMI score had a good discrimination between the patients with different risk levels, and the cumulative survival rate had a log rank *p* value < 0.01 for patients with different risk levels, while the difference in the cumulative survival rate between patients with different risk levels in the HEART score was not statistically significant (*p* > 0.05) (Figure 4a).

At the end of follow-up, in the GRACE score the mortality rate was 5% in the low-risk patients, 11.5% in the intermediate-risk patients, and 27.8% in the high-risk patients. The risk of death was significantly higher in the intermediate-risk patients and high-risk patients than in the low-risk patients (HR = 2.402, 95% CI: 1.371–4.208, *p* < 0.01; HR = 6.339, 95% CI: 3.783–10.622, *p* < 0.01). In the TIMI score, the mortality rate was 8.6% in the low-risk patients, 20% in the intermediate-risk patients, and 33.3% in the high-risk patients. The risk of death was also significantly higher in the intermediate-risk patients and high-risk patients than in the low-risk patients (HR = 2.423, 95% CI: 1.769–3.317, *p* < 0.01; HR = 4.496, 95% CI: 3.261–6.198, *p* < 0.01). In the HEART score, the mortality rate was 15% for the low-risk patients, 18.8% for the intermediate-risk patients, and 21% for the high-risk patients. There was no statistically significant difference in the risk of death between the intermediate-risk and the high-risk patients compared with the low-risk patients (HR = 1.340, 95% CI: 0.330–5.434, *p* > 0.05; HR = 1.524, 95% CI: 0.380–6.119, *p* > 0.05).

In the subgroup analysis of STEMI and NSTEMI patients, the HEART score could also distinguish patients with different risk levels (*p* < 0.01). (Figure 4b) Further analysis revealed that NSTEMI patients with an intermediate-risk had a higher death risk than the STEMI patients with a high-risk score (HR = 1.407, 95% CI:1.103–1.794, *p* < 0.01) in the HEART score.

**Figure 5:**
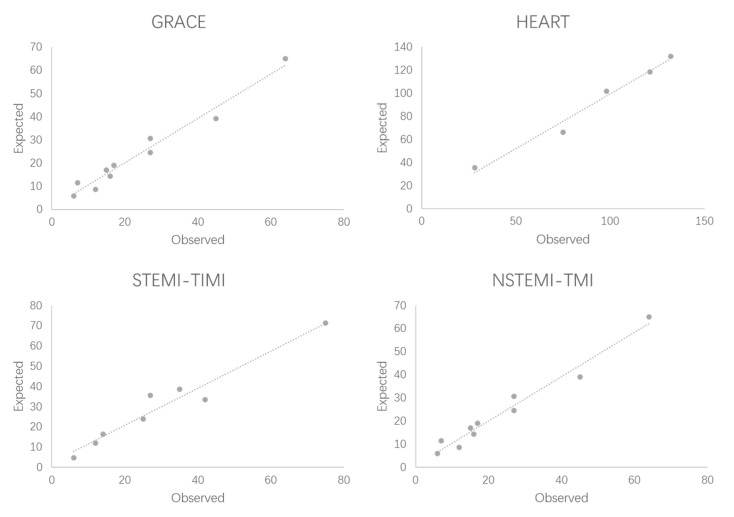
Hosmer–Lemeshow test for risk models.

Finally, the Hosmer–Lemeshow test showed that the *p*-values of the GRACE score, STEMI-TIMI score, NSTEMI TIMI score, and HEART score were 0.644, 0.363, 0.308, and 0.320, respectively. This means that the four risk models have a good degree of calibration. (Figure 5)

## 4. Discussion

The mortality of AMI patients is influenced by many factors, and the prognosis of patients with different levels of risk often varies greatly. Using tools such as a risk score to stratify risk in AMI patients can help to develop clinical strategies, but previous risk scoring models have focused on the short-term prognosis of patients, whereas several data show that it is equally important to assess the long-term prognosis of AMI patients. Although some risk scoring models have been developed for the long-term prognosis of AMI patients, they are still rare in the literature. Assessing the value of existing risk scoring models that have been widely used in the clinic in predicting the long-term prognosis of AMI patients may be a more convenient and rapid approach.

In this study, we found that the three most widely used risk scoring models, the GRACE score, TIMI score, and HEART score, can all predict the long-term prognosis of patients with AMI through an 8-year follow-up. Among the three risk scores, the GRACE score had the best prediction accuracy. In terms of risk stratification, both the GRACE score and the TIMI score were able to distinguish AMI patients with different risk levels, while the HEART score was only able to distinguish STEMI or NSTEMI patients with different risk levels in the subgroup analysis and performed poorly for overall AMI patient stratification. Moreover, all risk models passed the Hosmer–Lemeshow test.

There are only a few reports on the use of the GRACE score and TIMI score to predict the long-term prognosis of AMI patients, and the results of the studies are inconsistent. In one study, the AUC values of the GRACE score for predicting 3-year mortality in patients with STEMI or NSTEMI were 0.77 and 0.78, respectively, and both were higher than the TIMI score (AUC = 0.68 and 0.69, both *p* < 0.01) [23]. Another study that included only STEMI patients reported similar results, with the GRACE score being more accurate than the TIMI score in predicting the 3-year mortality in STEMI patients (0.77 versus 0.66, *p* < 0.01) [24]. However, some studies have also shown that the GRACE score is less accurate than the TIMI score in predicting 1-year mortality in STEMI patients (0.47 versus 0.75, *p* < 0.01) [25]. The advantage of the GRACE score over the TIMI score in predicting 1- and 5-year mortality was also not found in the study by Kozieradzka (0.81 versus 0.81, *p* > 0.05; 0.74 versus 0.73, *p* > 0.05) [26]. However, the former of the latter two studies excluded patients with cardiogenic shock and cardiac arrest, and the latter included only patients who underwent emergency PCI, which may not reflect the full picture of STEMI patients. Our study has the largest number of participants and longest follow-up to date, includes both STEMI and NSTEMI patients, and does not specifically exclude patients, which can best reflect the true performance of the risk scoring model. The AUC of the GRACE score for predicting the 1-year mortality was 0.78 for both STEMI and NSTEMI patients, and although the AUC value declined with longer follow-up, it also reached 0.73 until the end of follow-up (approximately 8 years). In contrast, the TIMI score was close to the GRACE score only at year 1, and the AUC values were lower than the GRACE score at 3 years, 5 years, and up to the end of follow-up. Therefore, the GRACE score was superior to the TIMI score in predicting long-term mortality.

There is only one report on the HEART score in predicting long-term prognosis in patients with ACS, which showed that patients with a score ≥ 4 had a much higher mortality rate than those with a score ≤ 3 at 5 years of follow-up (48.2% versus 10.6%, *p* < 0.01), but the AUC value of the HEART score in predicting the long-term mortality was not given in their study [15]. Our results showed that the HEART score was equally predictive of distant prognosis in STEMI patients and NSTEMI patients (AUC value > 0.5), but the predictive accuracy was inferior to both the GRACE score and the TIMI score. This result is not difficult to understand, as the HEART score is an emergency score for patients with chest pain whose diagnosis is still unclear. Its primary aim is to distinguish patients that are at high-risk in the short-term as soon as possible, so applicability and simplicity come first, and some data that are less readily available during the emergency period are then excluded [12]. Meanwhile, the assessment of the degree of suspicious medical history in the HEART score is somewhat subjective, which may also affect its predictive accuracy.

The higher accuracy of the GRACE score among the three scores may be related to some unique indicators. For example, creatinine has been previously shown to be a risk factor for long-term cardiovascular complications and long-term mortality in patients with AMI. At eGFR below 81 mL/1.73 min*m^2^, the risk of death and nonfatal cardiovascular outcomes in patients rises by 10% for every 10 unit decrease in eGFR (RR = 1.1, 95% CI:1.08–1.12) [27]. The Killip classification is also a factor that is associated with long-term prognosis in AMI patients. A higher Killip classification of AMI patients tends to have a combination of more severe coronary lesions and larger infarct size, which implies more myocardial cell necrosis, and the necrotic cells are subsequently replaced by fibrotic scars. Fibrous scar formation is difficult to reverse afterwards and has a range of adverse effects on the long-term prognosis of patients through its effect on cardiac contractility and interference with normal cardiac electrical activity leading to arrhythmias [28,29,30,31]. Although the TIMI score for STEMI patients includes the Killip classification, the model for NSTEMI patients does not include this factor, which may be one of the reasons why the TIMI score has a higher predictive accuracy for STEMI patients than for NSTEMI patients. In addition, the GRACE score treats risk factors such as age, heart rate, and blood pressure as continuous variables, which allows for a more refined risk assessment. In contrast, the TIMI score and HEART score consist of only dichotomous variables and provide limited feedback.

In terms of risk stratification, an interesting result is that the HEART score does not allow risk stratification of all AMI patients but performs well in the evaluation of STEMI patients or NSTEMI patients alone. This may be related to electrocardiographic performance as an evaluation metric. Typical ST-segment elevation accounts for 2 points in the HEART score, which is only a maximum of 10 points. This would result in lower scores for NSTEMI patients compared to STEMI patients, causing a proportion of high-risk NSTEMI patients to be assigned to the intermediate-risk group with a score of 7 or less and a proportion of intermediate-risk STEMI patients to the high-risk group. Thus, in the overall AMI grouping, patients in the intermediate-risk and high-risk groups are a mix of patients with both risk levels, resulting in poor stratification.

A clearer perception of the long-term prognosis of AMI patients may guide clinicians to make more effective interventions in clinical practice, while increasing patients’ perception of their own risk level may also increase their medication adherence to some extent. When assessing the short-term prognosis of patients with AMI, simplicity and ease of use may be a higher priority due to the need to determine the patient’s level of risk as soon as possible. However, when assessing the long-term prognosis of patients, the emphasis may be more on the accuracy of prediction. Therefore, the GRACE score, although more cumbersome than the TIMI score and HEART score, remains the score of choice for assessing the long-term prognosis of patients with AMI.

## 5. Limitations

First, only the GRACE score, TIMI score, and HEART score were compared in this study, and although many other risk scores are currently available, most of them are not widely used in clinical practice and were excluded. Second, because these data were not collected prospectively, the assessment of the degree of suspicion of medical history in the HEART score could only be calculated by a proxy for clinical suspicion of ACS, and the score results might have been different if the data had been collected prospectively. Finally, this study is a single-center retrospective study with a predominantly Chinese population, which may introduce some population bias.

## 6. Conclusions

In this study, a comparison of the GRACE score, TIMI score, and HEART score revealed that the GRACE risk score could represent a more accurate model to assess long-term death of acute myocardial infarction. The higher accuracy of the GRACE score may be related to its unique index and continuous variables, but further studies are required.

## Figures and Tables

**Table 1:** Risk Scores.

Variables		GRACE		TIMI (STEMI)	TIMI (NSTEMI)	HEART
Age		X		X	X	X
Medical history	Time from onset to visit			X		
	Suspicious degree					X
	Severe angina (≥2 events in last 24 h)				X	
	Use of aspirin last 7 days				X	
Physical examination	Body weight			X		
	Heart rate	X		X		
	Systolic blood pressure	X		X		
	Killip class	X		X		
ECG	ST deviation	X		X	X	X
	Repolarization disorder, BBB, or LVH			X		X
	Cardiac arrest at admission	X				
Laboratory results	Creatinine level	X				
	Troponin T level	X			X	X
Risk factors	Cardiovascular risk factors *			X	X	X
	Previous coronary artery disease (≥50%)				X	
	Previous revascularization					X
	History of cerebrovascular vascular disease					X
	History of peripheral vascular disease					X
	Obesity					X
Risk stratification	Low-risk	STEMI	<125	0–3	0–2	0–3
		NSTEMI	<108			
	Intermediate-risk	STEMI	126–154	4–6	3–4	4–6
		NSTEMI	109–140			
	High-risk	STEMI	>154	7–14	5–7	7–10
		NSTEMI	>140			

X: this risk factor is included in the risk score, ECG: electrocardiogram, BBB: bundle branch block, LVH: left ventricular hypertrophy, STEMI: ST-segment elevation myocardial infarction, NSTEMI: non-ST-segment elevation myocardial infarction. * Cardiovascular risk factors were defined as hypertension, diabetes, smoking, hypercholesterolemia, and family history of cardiovascular disease.

**Table 2:** Baseline characteristics of patients.

CLINICAL CHARACTERISTICS	TOTAL (N = 2220)	STEMI (N = 1494)	NSTEMI (N = 726)
**AGE, YRS**	66 (56, 74)	63 (53, 72)	70 (62, 75)
**MALE, N (%)**	1752 (78.9)	1220 (81.7)	565 (73.3)
**VITAL SIGNS AT PRESENTATION**			
**SYSTOLIC BLOOD PRESSURE, MMHG**	125 (110, 140)	122 (109, 138)	130 (113, 146)
**DIASTOLIC BLOOD PRESSURE, MMHG**	75 (66, 84)	74 (66, 85)	75 (66, 83)
**HEART RATE, BEATS/MIN**	78 (67, 88)	78 (68, 89)	77 (66, 86)
**KILLIP CLASS > II, N (%)**	1220 (55.0)	793 (53.1)	477 (58.8)
**CARDIAC RISK FACTORS, N (%)**			
**HYPERTENSION**	1099 (49.5)	657 (44.0)	468 (60.1)
**DIABETES**	449 (20.2)	261 (17.5)	199 (25.9)
**HYPERLIPIDEMIA**	69 (3.1)	47 (3.1)	22 (3.0)
**CURRENT SMOKING**	1358 (61.2)	1026 (65.0)	388 (53.4)
**OBESITY (BMI ≥ 28 KG/M2)**	307 (13.8)	205 (13.7)	102 (14.0)
**POSITIVE FAMILY HISTORY**	103 (4.6)	62 (4.1)	41 (5.6)
**HISTORY OF CARDIOVASCULAR DISEASE**	270 (12.2)	119 (8.0)	151 (20.8)
**HISTORY OF AMI**	256 (11.5)	173 (11.6)	83 (11.4)
**HISTORY OF PCI**	33 (1.5)	13 (0.9)	20 (2.8)
**HISTORY OF CABG**	5 (0.2)	1 (0.1)	4 (0.6)
**HISTORY OF CVA/TIA**	98 (4.4)	63 (4.2)	35 (4.8)
**HISTORY OF PERIPHERAL ARTERIAL DISEASE**	11 (0.5)	4 (0.3)	7 (1.0)
**LABORATORY RESULTS AT PRESENTATION**			
**MEAN CREATININE IN μMOL/L (SD)**	97 (73,103)	84 (72,100)	90 (75, 111)
**CTNT, NG/L**	1702 (343, 4025)	2440 (593, 4801)	743 (177, 2212)
**NT-PROBNP, PG/ML**	1661 (646, 4222)	1714 (671, 4142)	1586 (566, 4466)
**MEDICATION AT PRESENTATION, N (%)**			
**ASPIRIN**	2046 (92.2)	1390 (93.0)	656 (90.3)
**CLOPIDOGREL/TICLOPIDINE**	2099 (94.5)	1411 (94.4)	688 (94.8)
**ACEI/ARB**	1183 (53.3)	783 (52.4)	398 (54.8)
**BETA BLOCKERS**	1424 (64.1)	963 (64.5)	461 (63.5)
**STATINS**	2033 (91.6)	1360 (91.0)	673 (92.7)
**FOLLOW-UP AND ENDPOINT EVENTS**			
**FOLLOW-UP TIME, YEARS**	8 (7.3, 8.9)	8.1 (7.4, 9)	7.9 (6.8, 8.8)
**1-YEAR CUMULATIVE DEATH, N (%)**	64 (2.9)	30 (2.0)	34 (4.7)
**3-YEAR CUMULATIVE DEATH, N (%)**	152 (6.8)	79 (5.3)	73 (10.1)
**5-YEAR CUMULATIVE DEATH, N (%)**	259 (11.7)	135 (9.0)	124 (17.1)
**CUMULATIVE DEATHS AT THE END OF FOLLOW-UP, N (%)**	454 (20.5)	236 (15.8)	218 (30.0)

mmHg: millimeters of mercury, BMI: body mass index, AMI: acute myocardial infarction, PCI: percutaneous coronary intervention, CABG: coronary arterial bypass grafting, CVA: cerebrovascular attack, TIA: transient ischemic attack.

**Table 3:** Comparison of the AUC values for the three risk scores at different time points *.

Cumulative Deaths	GRACE	TIMI	HEART	*p*		
				G vs. T	G vs. H	T vs. H
STEMI						
1-year	**0.775**	0.770	0.719	0.87	0.25	0.29
3-year	0.762	0.719	0.643	<0.05	<0.01	<0.05
5-year	0.740	0.702	0.617	<0.05	<0.01	<0.01
End of follow-up	0.734	0.675	0.632	<0.01	<0.01	<0.05
NSTEMI						
1-year	0.779	0.730	0.739	0.30	0.37	0.88
3-year	0.762	0.678	0.658	<0.05	<0.01	0.58
5-year	0.738	0.656	0.629	<0.01	<0.01	0.30
End of follow-up	0.730	0.665	0.611	<0.01	<0.01	<0.01

* G: GRACE score, T: TIMI score, H: HEART score.

## Data Availability

The datasets used and/or analyzed during the current study are available from the corresponding author on reasonable request.

## Abbreviations

ACS: acute coronary syndrome; AMI: acute myocardial infarction. BBB: bundle branch block. BMI: body mass index. CABG: coronary arterial bypass grafting. CVA: cerebrovascular attack. LVH: left ventricular hypertrophy. MACEs: major adverse cardiac events. NSTEMI: non-ST-segment elevation myocardial infarction. PCI: percutaneous coronary intervention. STEMI: ST-segment elevation myocardial infarction. TIA: transient ischemic attack.
